# Full-thickness skin burn complicated by high-power radiofrequency ablation of persistent atrial fibrillation

**DOI:** 10.1016/j.hrcr.2023.12.019

**Published:** 2024-01-05

**Authors:** Tushar Pansuriya, Sajjad Gul, Mustafa Hassan

**Affiliations:** Department of Cardiovascular Medicine, Michigan State University- McLaren Hospital, Flint, Michigan

**Keywords:** Catheter ablation, Atrial fibrillation, Skin burn, High-power radiofrequency, Indifferent electrode, Grounding patch

**Key Teaching Points**

• High-power short-duration catheter ablation has demonstrated superiority in terms of efficacy, reduced complication rates, and shorter procedure times compared to conventional techniques, contributing to a decreasing overall complication trend in the field.
• Skin burns at indifferent electrode sites, though rare, are significant complications in radiofrequency catheter ablation. Implementing meticulous procedural protocols, including optimal electrode placement, uniform heat dispersion, and attention to factors like subdermal fat and attachment quality, is crucial for preventing such complications.
• This case highlights the need for ongoing research and heightened awareness in the field of electrophysiology to ensure the safe application of indifferent electrodes. Understanding contributing factors and promptly addressing complications through proper electrode attachment and postprocedural examination are critical for enhancing patient safety and outcomes.

## Introduction

High-power short-duration (HPSD) radiofrequency (RF) catheter ablation emerged in 2006 and has recently become the primary modality for RF catheter ablation for atrial fibrillation (Afib).^1^^,^^2^ Extensive studies have demonstrated its superior efficacy, reduced procedure time, and comparable complication rates when compared to conventional ablation methods.^1^, ^2^, ^3^, ^4^ Indifferent electrodes are used in RF ablation techniques to disperse heat energy from the body.^5^^,^^6^ Although skin burns at indifferent electrodes are theoretically possible, they remain rare complications of RF catheter ablation. We present a rare case of full-thickness skin burn related to HPSD RF ablation for persistent Afib.

## Case report

This case concerns a 68-year-old female patient with a history of persistent Afib, morbidly obese (body mass index of 38), who had previously undergone 2 catheter ablation procedures. Initially, 5 years ago, she received cryoballoon pulmonary vein isolation, left atrial posterior wall isolation, and a right-sided cavotricuspid isthmus ablation line. Two years later, the patient developed recurrent symptomatic Afib. She underwent further interventions with reisolation of the left superior and inferior pulmonary veins by RF ablation and ablation of mitral isthmus–dependent left-sided atrial flutter. Despite these efforts, she developed recurrent persistent Afib 2 years after second intervention, necessitating a third ablation procedure.

The third ablation procedure used the TactiCath™ Contact Force Ablation Catheter, Sensor Enabled™ (Abbott, Abbott Park, IL). The procedure involved RF ablation of reconnected right-sided pulmonary veins and left atrial posterior wall reisolation under general anesthesia support. A grounding patch was placed on the patient’s right lower back, and the dragging RF ablation technique was employed, applying an average power of 50 W for 10 seconds for each lesion. The RF ablation procedure was conducted with an average temperature of 32°C and an average impedance of 114 Ω. The total fluoroscopy time was 11 minutes, and the total RF ablation time was 25 minutes. Esophageal probe temperature monitoring was employed as per protocol.

**Figure 1:**
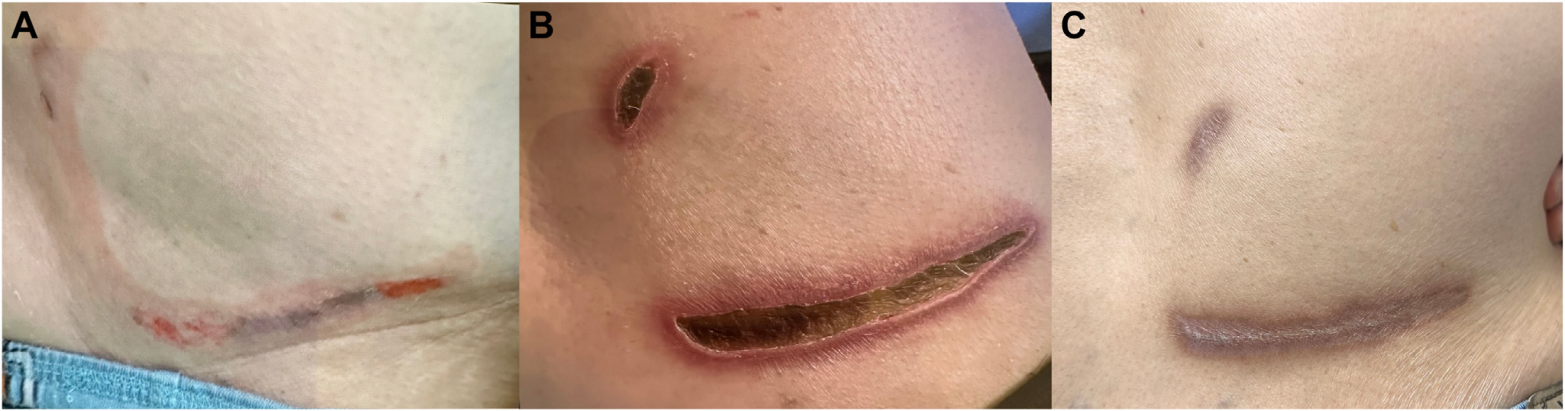
**A:** Second day post radiofrequency ablation; erythematous skin lesion was noted at indifferent electrode site at right lower back. **B:** Within 5 days, lesion developed into full-thickness skin burn. **C:** After 3 months, skin burn healed completely, with some residual scar.

Postprocedure, no immediate skin changes were noted at the grounding patch site. However, on the second day, the patient reported pain and observed skin redness at the right lower back (Figure 1A). Subsequent examination on day 5 revealed a full-thickness skin burn (Figure 1B). The burn was treated with silver sulfadiazine topical therapy, resulting in gradual improvement and complete healing in 3 months, leaving a residual scar (Figure 1C). Importantly, the patient maintained a normal sinus rhythm.

## Discussion

Several studies have confirmed the superior effectiveness and reduced procedure time associated with HPSD catheter ablation compared to conventional techniques. Moreover, HPSD catheter ablation has demonstrated noninferiority in complication rates.^1^, ^2^, ^3^, ^4^

RF catheter ablation involves using a catheter tip to ablate myocardial tissue, aided by an indifferent electrode, commonly known as a grounding or dispersive patch, placed on the skin to disperse RF heat energy.^5^^,^^6^ The extent of heat dispersion at the indifferent electrode site depends on various factors, including RF power, duration of RF use, electrode size, quantity and placement, attachment quality, and subdermal fat.^5^^,^^6^ Before the introduction of HPSD RF catheter ablation, lower-power RF methods were prevalent. Conventional-power RF delivers 25–35 W over 30–60 seconds, whereas high-power RF delivers 40–50 W over 5–10 seconds.^1^^,^^4^ The popularity of HPSD catheter ablation has increased, contributing to a significant decrease in overall complication rates owing to advancements in ablation technologies and increased clinical experience.^7^^,^^8^

Skin burns at indifferent electrode sites are severe and rare complications, with sparse documentation in the literature.^5^ Although cases of full-thickness skin burns secondary to tumor RF ablation have been reported, occurrences related to RF catheter ablation are notably infrequent. Although large studies did not report of skin burns in the context of RF ablation,^1^^,^^3^^,^^4^ isolated case reports exist, such as those documented by Dhillon and colleagues,^9^ highlighting 3 cases of skin burns at indifferent electrodes successfully treated with topical therapy or skin grafting interventions.

Given the rising trend of HPSD RF ablation, understanding and preventing this complication are paramount. The positioning of the indifferent electrode has the potential to impact both the efficacy of ablation and the impedance of the circuit.^10^^,^^11^ The literature emphasizes the importance of uniform heat dispersion at indifferent electrode sites to effectively mitigate the risk of localized heat density.^6^ A strategic approach advocated in the literature involves the utilization of multiple indifferent electrodes at the same level, which can be executed through either sequential activation or simultaneous activation of more than 1 electrode, demonstrating promise in minimizing localized heat density on the skin.^6^^,^^10^ Key factors identified in the literature as significant contributors to an elevated risk of skin burns during RF ablation procedures include improper electrode attachment, the application of a singular electrode, the presence of excessive subdermal fat at the site of the indifferent electrode, high-energy application, and prolonged durations of energy application.^5^ This comprehensive understanding of contributing factors is pivotal in shaping meticulous procedural protocols, reflecting the ongoing efforts within the clinical research community to refine catheter ablation methodologies and ultimately enhance patient safety and outcomes.

The skin burn observed in our case is likely attributed to the patient’s class 2 obesity, use of a single indifferent electrode, and possible malattachment of the indifferent electrode. The use of the dragging technique with HPSD RF ablation and relatively long RF duration may have contributed to this complication.

## Conclusion

Skin burns at indifferent electrodes during RF catheter ablation are rare yet significant complications. This case study underscores the imperative need for meticulous indifferent electrode implementation to prevent skin burns in the era of HPSD catheter ablation. Prompt recognition and prevention of such complications through proper electrode attachment and postprocedural examination are critical. Further research and heightened awareness are warranted to ensure the safe application of RF catheter ablation techniques within the field of electrophysiology.

## Disclosures

All authors have no conflicts to disclose.
